# Rutin Prevents Dexamethasone-Induced Muscle Loss in C2C12 Myotube and Mouse Model by Controlling FOXO3-Dependent Signaling

**DOI:** 10.3390/antiox12030639

**Published:** 2023-03-03

**Authors:** Young-Sool Hah, Won Keong Lee, Seung-Jun Lee, Sang Yeob Lee, Jin-Hee Seo, Eun Ji Kim, Yeong-In Choe, Sang Gon Kim, Jun-Il Yoo

**Affiliations:** 1 Department of Orthopedics, Institute of Health Sciences, Gyeongsang National University School of Medicine and Hospital, Jinju 52727, Republic of Korea; 2 Biomedical Research Institute, Gyeongsang National University Hospital, Jinju 52727, Republic of Korea; 3 Department of Convergence of Medical Sciences, Gyeongsang National University, Jinju 52828, Republic of Korea; 4 Crop Production Technology Research Division, National Institute of Crop Science, Rural Development Administration, Miryang 50424, Republic of Korea; 5 Anti-Aging Research Group, Gyeongnam Oriental Anti-Aging Institute, Sancheong 52215, Republic of Korea; 6 Department of Orthopedic Surgery, Inha University Hospital, Incheon 22332, Republic of Korea

**Keywords:** dexamethasone, muscle loss, sarcopenia, rutin, catabolism

## Abstract

One of the causes of sarcopenia is that homeostasis between anabolism and catabolism breaks down due to muscle metabolism changes. Rutin has shown antioxidant and anti-inflammatory effects in various diseases, but there are few studies on the effect on muscle loss with aging. The effect of rutin on muscle loss was evaluated using dexamethasone-induced muscle loss C2C12 myoblast and mouse model. In the group treated with dexamethasone, the muscle weight of gastrocnemius (GA), tibialis anterior (TA), and extensor digitorum longus (EDL) in the mouse model were significantly decreased (*p* < 0.0001 in GA, *p* < 0.0001 in TA, and *p* < 0.001 in EDL) but recovered (*p* < 0.01 in GA, *p* < 0.0001 in TA, and *p* < 0.01 in EDL) when treated with rutin. MAFbx, MuRF1, and FOXO3 protein expression of C2C12 myoblast were significantly increased (*p* < 0.01 in MAFbx, *p* < 0.01 in MuRF1, and *p* < 0.01 in FOXO3) when treated with dexamethasone, but it was recovered (*p* < 0.01 in MAFbx, *p* < 0.01 in MuRF1, and *p* < 0.01 in FOXO3) when rutin was treated. In addition, MAFbx and FOXO3 protein expression in GA of mouse model was significantly increased (*p* < 0.0001 in MAFbx and *p* < 0.001 in FOXO3) when treated with dexamethasone, but it was also recovered (*p* < 0.01 in MAFbx and *p* < 0.001 in FOXO3) when rutin was treated. The present study shows that rutin blocks the FOXO3/MAFbx and FOXO3/MuRf1 pathways to prevent protein catabolism. Therefore, rutin could be a potential agent for muscle loss such as sarcopenia through the blocking ubiquitin-proteasome pathway associated with catabolic protein degradation.

## 1. Introduction

Flavonoids are organic compounds with diverse phenolic compounds [1]. These compounds are known for their antioxidants by acting as scavengers for reactive oxygen species (ROS) and anti-inflammatory properties through various mechanisms such as inhibition of transcription factors involved in inflammatory pathways [1,2]. These characteristics are thought to be the cause of their potential positive impacts on a wide range of human disorders. They may help in reducing the risk of cardiovascular disease, cancer, neurodegenerative diseases such as Alzheimer’s and Parkinson’s, diabetes, osteoporosis, and age-related macular degeneration [3,4]. However, it is important to note that more research is needed to confirm these potential benefits and to understand the optimal dosage and duration of flavonoid intake. Some examples of flavonoid subclasses include flavonols, flavones, flavanones, isoflavones, anthocyanidins, and catechins. They can be found in many food sources such as fruits, vegetables, nuts, seeds, and common beverages such as tea, wine, and chocolate [5].

Rutin, one of the flavonoids, can be extracted from various types of fruits such as oranges, grapes, and lemons [6]. Rutin has been reported to have various therapeutic properties such as cerebrovascular diseases, antioxidants, and hepatoprotective properties [7,8,9]. Rutin also showed anti-inflammatory effects on various inflammatory conditions. The relevant study showed that rutin exhibits an anti-inflammatory effect in all 5, 10, 20, 50, and 100 μM in LPS-induced RAW 264.7 cells [10]. This result suggested that rutin could be a therapeutic agent for various disorders.

The loss of skeletal muscle mass must be caused by a negative protein balance, and the histology evidence suggests that this loss is caused by both a reduction in myocyte numbers and size caused by protein degradation [11]. The pathway associated with decreased muscle protein includes the ubiquitin-proteasome pathway [12]. This mechanism is known to be related to muscle ring-finger protein 1 (MuRF1) and the muscular atrophy F-box (MAFbx), which are inhibited by insulin-like growth factor-1 (IGF-1) [13,14]. In addition, MuRF1 and MAFbx are involved in FOXO signaling, one of the transcription factors required for disuse muscle atrophy [15,16].

MuRF1, a protein that is involved in tagging other proteins with ubiquitin for degradation, is linked to muscle wasting and weakness in conditions such as aging and cancer cachexia [17]. MAFbx, another protein that plays a role in muscle protein degradation, also contributes to muscle wasting and weakness in certain conditions [18]. FOXO signaling, which is controlled by FOXO proteins, also plays a role in muscle mass and function by regulating muscle protein degradation. FOXO3 belongs to a family of forkhead transcription factors and is involved in a wide range of physiological processes. When activated, FOXO3 can lead to muscle protein degradation by inducing the expression of MuRF1 and MAFbx [19]. Therefore, by regulating muscle protein degradation, MuRF1, MAFbx, and FOXO signaling are all closely related to muscle mass and function, and their increased levels have been associated with muscle wasting and weakness.

Recently, studies have also explored the connection of rutin with the loss of muscle mass. Current research on the relationship between rutin and muscle loss is still in its infancy, with a limited number of studies being carried out. For instance, a study on rats demonstrated that rutin supplementation improved muscle mass, strength, and function, and decreased muscle damage in a dose-dependent manner [20]. Another study on rats showed that rutin supplementation improved muscle mass and function, and reduced inflammation and oxidative stress in a model of sarcopenia caused by ovariectomy [21]. Therefore, current research suggests that rutin may have a beneficial effect on muscle loss by enhancing muscle mass and function and reducing muscle damage, inflammation, and oxidative stress. However, further studies are required to confirm these findings and to determine the optimal dosage, duration of rutin supplementation, and potential molecular pathway

Rutin has shown antioxidant and anti-inflammatory effects in various diseases, but there are few studies on the effect on muscle loss with aging [8,9]. Therefore, the present study aims to examine the efficacy of rutin in aging-induced muscle loss by applying rutin to dexamethasone-induced muscle loss cells and animal models.

## 2. Materials and Methods

### 2.1. Cell Experiment Using Mouse C2C12 Myoblast

To evaluate the effect of rutin on the muscle loss prevention effect, a cell experiment using mouse C2C12 myoblasts (Manassa, VA, USA) was performed. C2C12 myoblasts were cultured 1 × 10^5^ cells/mL in each 6-well with 90% Dulbeco’s modified Eagle’s medium (DMEM), 10% fetal bovine serum (FBS), and 100 units/mL of penicillin-streptomycin (PS) (15140122; Thermofisher Scientific, Waltham, MA, USA). After the cells reached 90% confluence, the medium was replaced with a differentiation medium containing 2% horse serum and 100 units/mL PS. The differentiation medium was applied for a total of 7 days and was replaced every 2 days. The differentiated cells were divided into four groups as follows: control group (CTR), dexamethasone treatment group (Dexa), rutin treatment group (rutin), and dexamethasone + rutin treatment group (DR). To make a muscle loss model, dexamethasone (1 uM) was applied to the DEXA group and DR for 48 h, and rutin (100 µM) was additionally applied to the rutin group and the DR group for the same period. All experiments were conducted as multiple experiments.

### 2.2. Cell Viability Analysis

Cell viability analysis was performed according to the instructions of the Cell Counting Kit-8 (Dojindo Laboratories, Kumamoto, Japan). Specifically, concentrations of 0, 0.25, 0.5, 1, and 2 µM of rutin were added to C2C12 cells seeded at 1 × 10^5^ cells/mL in 24-well plates and cultured for 24 h. After that, 10 µL of CCK-8 was added and incubated at 37 °C for one hour, and the formazan dye produced at 450 μm was measured using a microplate reader (Molecular Devices, San Jose, CA, USA).

### 2.3. Animal Experiments Using C57BL/6 Mice

C57BL/6 mice (Core Tech Co., Ltd. in Seoul, Korea) with an average weight of 22 g at 6 weeks of age were used for animal experiments. Mice had the adaptation period in a light and dark cycle for a week at temperature of 24 ± 2 °C, 40–60% relative humidity, 150–300 lux lighting, and a 12 h interval. Mice were supplied with regular food and fed with freely provided sterile water. After the adaptation period, the mice were divided into three groups (n = 9 per group): a control group (CTR), a dexamethasone treatment group (Dexa), and a dexamethasone + rutin treatment group (DR).

After the adaption phase, the Dexa and DS groups received intraperitoneal injections of dexamethasone (20 mg/kg of body weight of mice) at 10~11 a.m. every day for two weeks to make a muscle loss mouse model, whereas the CTR group received saline injections in the same period. In the DR group, rutin (Glentham Life Sciences, Wiltshire, United Kingdom) was orally administered at an amount of 60 mg/kg body weight of mice once daily from 1 week before administration of dexamethasone until the end of the experiment. On the last day of the experiment, the gastrocnemius (GA) muscle, tibialis (TA) anterior, and extensor digitorum longus muscle from each group were weighed. In addition, GA muscle and TA muscle from each group were immediately frozen in liquid nitrogen and maintained at −80 °C.

### 2.4. Histological Analysis of Muscle Tissue

Cryosectioning of the right TA muscle and GA muscle was performed using Optimal Cutting Temperature (OCT) compounds (Lab-Tek; Miles Laboratories, Inc., Naperville, IL, USA) after instantly freezing the samples. Muscle slices 5 mm thick were cut from the frozen samples using a cryostat (Leica CM1950; Heidelberg, Germany). To block nonspecific binding, the slices were incubated for 1 h at room temperature in 10 percent goat serum. Wheat germ agglutinin, Alexa Fluor488 conjugate (W11261; Invitrogen/Thermo Fisher Scientific, Waltham, MA, USA) antibody was diluted to a concentration of 1:500, and the slices were stained at 4 °C overnight. The extracellular matrix was observed using an upright microscope (Nikon Eclipse ni DSRi2; Nikon, Tokyo, Japan). Fiber cross-sectional area (CSA) and Min Feret diameter were measured using ImageJ application after imaging the samples at 100 magnification with a microscope.

### 2.5. Western Blot Analysis

The samples of cells and tissues were washed with cold phosphate buffered saline (PBS) buffer. Radioimmunoprecipitation assay buffer (RIPA, 10 mM Tris-Cl, pH 7.4, 150 mM NaCl, 1 mM EDTA, 1% Triton X-100, 1% sodium deoxycholate, 0.1 percent SDS) which is supplemented with phenylmethylsulfonyl fluoride (PMSF), protease inhibitor cocktail, and sodium orthovanadate (Santa Cruz Biotechnology, Santa Cruz, CA, USA) were used to lyse the samples for 10 min. After homogenization of the sample with an ultrasonic grinder, centrifugation was performed at 13,000× *g* for 15 min at 4 °C and quantified with BCA Protein Assay Kit (Pierce, Rockford, IL, USA). The quantified protein was subjected to electrophoresis on 10% SDS polyacrylamide gel by 30 µg total protein and transferred to a nitrocellulose membrane. Each blot was blocked with Tris-buffered saline containing 5% skim milk (Difco, Detroit, MI, USA) and 0.05 percent Tween 20 (TBST) at room temperature for 1 h. Each blot was incubated against primary antibodies of MuRF-1 (sc-398608; Santa Cruz Biotechnology, Dallas, TX, USA), MAFbx (sc-166806; Santa Cruz Biotechnology, Dallas, TX, USA), FOXO1 (2880s; Cell Signaling Technology, Danvers, MA, USA), FOXO3 (2497s; Cell Signaling Technology), Gapdh (ATGA0394; ATGen, Montevideo, Uruguay), and β-actin (A5441; Sigma-Aldrich, St. Louis, MO, USA) at 4 °C overnight. The next day, the membrane was washed 3 times with TBST and reacted with a peroxidase-conjugated secondary antibody. Washed 3 times again with TBST, each band was detected with Clarity Western ECL Substrate (Bio-Rad Laboratories, Inc., Berkeley, CA, USA) in a ChemiDoc™ Touch Imaging System (Bio-Rad Laboratories, Inc., Hercules, CA, USA). The intensity of the specific band in the image was quantified using densitometry (ImageJ software; NIH).

### 2.6. Statistical Analysis

Every experiment was performed three times. For statistical analysis, GraphPad Prism (Version 5.01; GraphPad Software, San Diego, CA, USA) was used and the results were expressed as mean ± SD or mean ± SE. When comparing two groups, Student’s *t*-test was used, and when comparing three or more groups, one-way ANOVA analysis was used with *p*-value of 0.05 for significant results.

## 3. Results

### 3.1. Evaluation of Rutin in the Muscle Loss Model of C2C12 Myotube Induced by Dexamethasone

**Figure 1:**
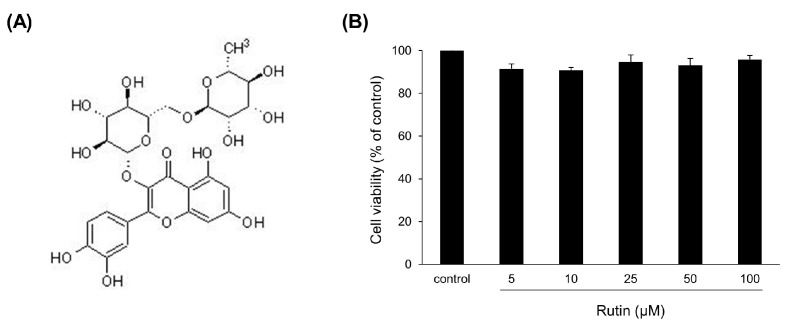
Effects of rutin on cell viability of C2C12 myoblasts. (**A**) The chemical structure of rutin. (**B**) C2C12 myoblasts were treated with different concentrations of rutin (5, 10, 25, 50, and 100 μM) for 24 h. Cell viabilities were determined using CCK-8 reagent. Values are expressed as percentages of the vehicle-treated control.

There was no significant difference in cell viability analysis with rutin (Figure 1A) at concentrations of 0.25, 0.5, 1, and 2 mM in C2C12 myotube (Figure 1B).

**Figure 2:**
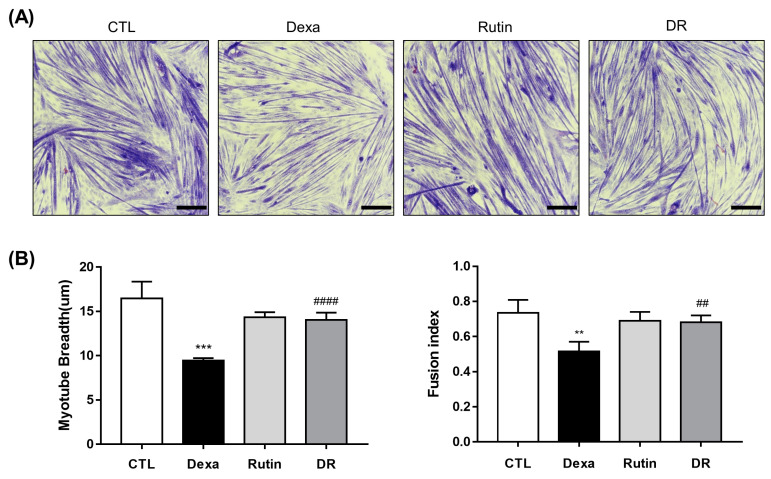
The preventive effect of rutin on dexamethasone-induced C2C12 myotube atrophy. (**A**) May–Grunwald and Giemsa staining of C2C12 cells. The scale bar represents 250 μm. (**B**) Quantification of myotube breadth and fusion index from May–Grunwald and Giemsa stained images. Data shown are mean ± S.D (n = 100 in myotube breadth measurement). ** *p* < 0.01 vs. CTL, *** *p* < 0.001 vs. CTL, ## *p* < 0.01 vs. Dexa. #### *p* < 0.0001 vs. Dexa. Dexa, Dexamethasone; DR, dexamethasone + rutin.

C2C12 myoblasts of each group were differentiated into myotubes, and myotube breath was measured for each group (Figure 2A). In the Dexa group, myotube breath was significantly decreased compared to the CTL group. However, it was significantly increased in the DR group compared to the Dexa group (Figure 2B). 

**Figure 3:**
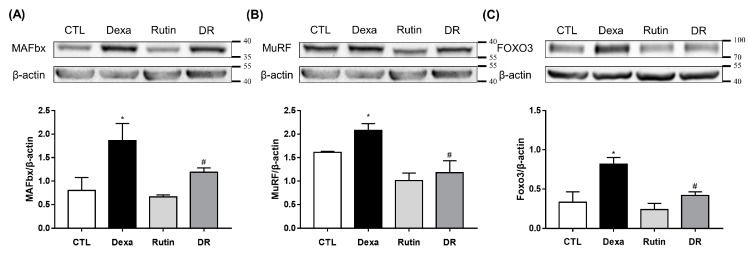
Rutin inhibits the protein expression of MAFbx, MuRF1, and FOXO3. C2C12 myotubes were treated with 100 μM rutin in the presence or absence of 1 μM Dexa for 48 h. (**A**) Western blot results of MAFbx and comparison of MAFbx protein expression between each group. (**B**) Western blot results of MuRF1 and comparison of MuRF1 protein expression between each group. (**C**) Western blot results of FOXO3 and comparison of FOXO3 protein expression between each group. * *p* < 0.05 vs. CTL, # *p* < 0.05 vs. Dexa. Dexa, Dexamethasone; DR, dexamethasone + rutin.

Additionally, the C2C12 myotube’s protein expression of MuRF1, MAFbx, and FOXO3 was evaluated. MAFbx protein expression was significantly increased in the Dexa group compared to the CTL group, but the DR group was significantly decreased compared to the Dexa group (Figure 3A). MuRF1 was significantly increased in the Dexa group compared to the CTL group and significantly decreased in the DR group compared to the Dexa group (Figure 3B). The protein expression of FOXO3 was also significantly increased in the Dexa group compared to the CTL group and decreased significantly in the DR group compared to the Dexa group (Figure 3C).

### 3.2. Prevention Effect of Rutin on Dexamethasone-Induced Muscle Loss Mouse Model

**Figure 4:**
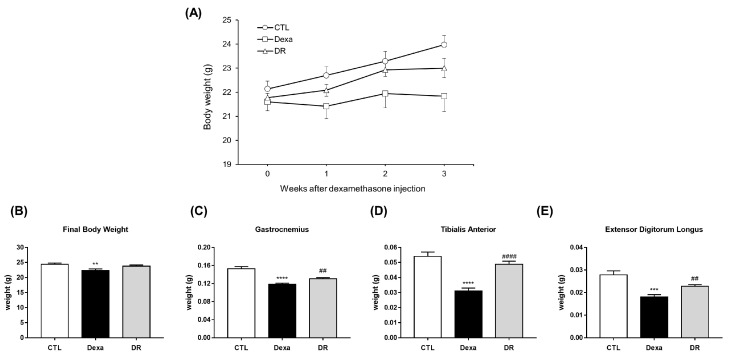
Rutin prevents muscle loss in the dexa-induced atrophy mouse model. (**A**) Body weight. (**B**) Comparison of final body weight in each group. ** *p* < 0.01 vs. CTL. (**C**) Muscle weight of gastrocnemius. **** *p* < 0.0001 vs. CTL. ## *p* < 0.01 vs. Dexa. (**D**) Muscle weight of tibialis anterior. **** *p* < 0.0001 vs. CTL. #### *p* < 0.0001 vs. Dexa. (**E**) Muscle weight of extensor digitorum longus. *** *p* < 0.001 vs. CTL. ## *p* < 0.01 vs. Dexa. Dexa, Dexamethasone; DR, dexamethasone + rutin.

Body weights were measured for 3 weeks in the control, DEXA, and DR groups (Figure 4A). In the final body weight, the Dexa group was significantly decreased in the CTL group. The DR group increased body weight compared to the DEXA group, but there was no significant difference (Figure 4B). The weights of each group for GA, TA, and EDL were compared. GA weight was significantly decreased in the DEXA group compared to the CTL group and significantly increased in the DR group compared to the DEXA group (Figure 4C). TA weight was significantly decreased in the DEXA group compared to the CTL group and significantly increased in the DR group compared to the DEXA group (Figure 4D). EDL weight was also significantly decreased in the DEXA group compared to the CTL group and significantly increased in the DR group compared to the DEXA group (Figure 4E). 

**Figure 5:**
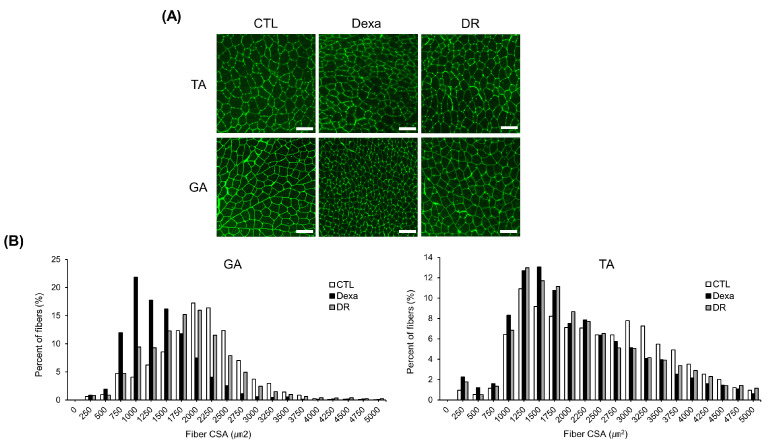
Rutin attenuates dexamethasone-induced muscle fiber area reduction in mice. (**A**) Representative images of immunofluorescent staining of myofiber cross-section of TA and GA. A microscope with a 10× objective was used to capture the images. The scale bar represents 100 μm. (**B**) Quantification of myofiber size by cross-sectional area (CSA) measurements for GA and TA muscle. Data are shown as mean ± S.E. (n = 9). GA, gastrocnemius muscles; TA, tibialis anterior; Dexa, Dexamethasone; DR, dexa + rutin.

In Figure 5A, a representative immunofluorescent staining of the myofiber cross-section of TA and GA is shown. In GA, fiber CSA was decreased in the DEXA group compared to the CTL group, but increased again in the DR group. Fiber CSA of TA also showed similar results among the three groups (Figure 5B). 

**Figure 6:**
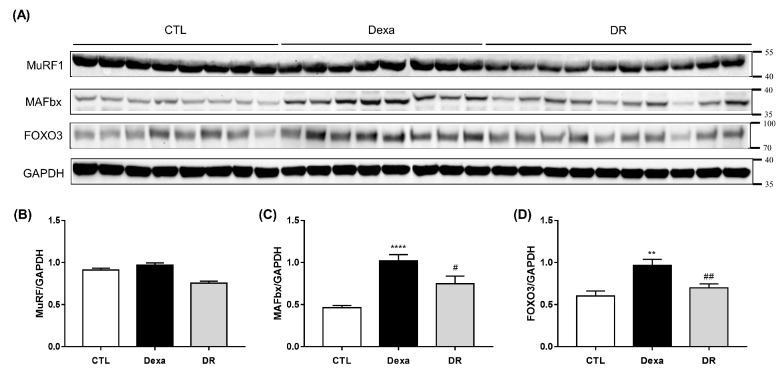
Rutin inhibits muscle loss in dexamethasone-induced atrophy mouse model via FOXO3-dependent signaling. (**A**) Western blot results of MAFbx, MuRF1, and FOXO3 in gastrocnemius. (**B**) Comparison of MAFbx protein expression between each group. (**C**) Comparison of MuRF1 protein expression between each group in gastrocnemius. (**D**) Comparison of FOXO3 protein expression between each group in gastrocnemius. ** *p* < 0.001, **** *p* < 0.0001 vs. CTL. # *p* < 0.05, ## *p* < 0.01 vs. Dexa. Dexa, Dexamethasone; DR, dexamethasone + rutin.

Since MuRF1 and MAFbx are known to be up-regulated by the catabolic pathways of skeletal muscle, we evaluated the protein expression of MuRF1 and MAFbx with associated transcription factors and FOXO3 in each group (Figure 6A). As a result, the protein expression of MAFbx was significantly increased in the Dexa group compared to the CTL group (Figure 6B). There was no significant difference in MuRF1 (Figure 6C). Moreover, FOXO3 was significantly increased in the Dexa group compared to the CTL group and significantly decreased in the DR group compared to the Dexa group (Figure 6D).

## 4. Discussion

Rutin may have a potential role in preventing or treating sarcopenia, a condition characterized by age-related loss of muscle mass and strength. This may be achieved through its interaction with the protein, MuRF1. MuRF1, a ubiquitin ligase, has been linked to muscle wasting and weakness in conditions such as cancer cachexia and aging. Studies in mice and rats have shown that rutin supplementation reduces the levels of MuRF1 leading to an increase in muscle mass and strength, and a decrease in muscle damage and inflammation [22]. Rutin’s interaction with MAFbx is similar to its interaction with MuRF1. Both proteins, MAFbx and MuRF1, are ubiquitin ligases and are involved in muscle protein degradation. However, unlike MuRF1, studies have found that rutin supplementation not only decreases the levels of MAFbx in muscle tissue but also reduces the protein’s ubiquitin ligase activity, leading to increased muscle mass and strength and decreased muscle damage and inflammation [23,24].

Rutin may have the potential to prevent or treat sarcopenia by interacting with the FOXO family of transcription factors. The FOXO family plays a role in regulating muscle mass and function by controlling muscle protein degradation. Studies in animals have suggested that rutin supplementation can decrease the activity of FOXO family members, leading to increased muscle mass and strength. Additionally, rutin may also have the ability to inhibit the activity of FOXO proteins by blocking their interactions with other activating proteins. However, it is worth noting that most of the research on rutin and FOXO proteins have been conducted in animals, and more research is needed to confirm these findings in humans [22,25].

Regulation of muscle mass and function is a key role played by the FOXO family of transcription factors. FOXO3 protein, in particular, has been seen to regulate the balance between muscle growth and muscle loss by promoting activation of genes involved in muscle protein synthesis and inhibiting genes involved in muscle protein degradation. Furthermore, FOXO3 also regulates genes involved in glucose metabolism and energy balance [26,27]. Catabolic activity in muscle refers to a breakdown of stored energy molecules such as glycogen and muscle proteins to provide energy for muscle contraction and other metabolic processes during times of stress such as exercise or fasting [28]. This process is regulated by different signaling pathways including insulin signaling pathway and AMPK pathway. FOXO proteins play a role in inhibiting insulin and insulin-like factor (IGF-1), and FOXO3 regulates glucose in skeletal muscle through transcriptional activity [27,29]. In addition, FOXO3 plays a key role in regulating catabolic activity in muscle by promoting activation of genes involved in glucose metabolism and energy balance, as well as inhibition of genes involved in muscle protein degradation [30].

The present study discovered that rutin treatment in a dexamethasone-induced muscle loss model had a therapeutic effect. In the C2C12 cell experiment, the myotube breadth of the Dexa group was significantly decreased compared to the control group but increased significantly again in the DR group. In the dexamethasone-induced muscle loss mouse model, the weight of skeletal muscles such as GA, TA, and EDL decreased significantly in the DEXA group compared to the control group but increased significantly in the DR group. In addition, fiber CSA and Minferet of GA decreased in the DEXA group compared to CTL group but increased in the DR group similarly to the CTL group. These results show that rutin has a muscle loss prevention effect, and several previous studies have also suggested a therapeutic effect of rutin on muscle [20,31].

In protein expression analysis using C2C12 myoblast, MAFbx, MuRF1, and FOXO3 significantly increased in the Dexa group compared to the CTL group, then significantly decreased again in the DR group treated with rutin. Moreover, in the dexamethasone-induced muscle loss mouse model, the protein expression of MAFbx and FOXO3 was significantly increased in the Dexa group compared to the CTL group, and there was no significant difference in the DR group, but there was a slight decrease. The results suggested that the ubiquitin-proteasome pathway is responsible for expression of these protein changes [19]. Murf1 and MAFbx are also known to be involved in the antioxidant defense of muscle cells, and both induce oxidative damage by increasing the production of reactive oxygen species (ROS). Studies suggest that Murf1 and MAFbx damage the mitochondrial function of muscle cells and induce muscle atrophy [32,33]. However, the present study showed that rutin reduced Murf1 and MAFbx expression in muscle. Therefore, rutin is thought to play a protective role against Murf1- and MAFbx-induced oxidative damage. 

**Figure 7:**
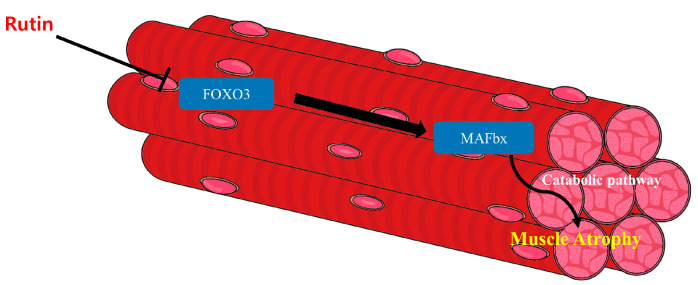
The overall rutin mechanisms related to muscle atrophy. Rutin down-regulates the transcriptional factor, FOXO3. Down-regulated FOXO3 becomes unable to affect the expression of MAFbx. Consequently, inhibited MAFbx cannot induce muscle atrophy via the catabolic pathway.

Since skeletal muscle accounts for the majority of the body’s overall mass, and catabolism is highly associated with maintaining skeletal muscle protein, catabolic stimulation is one of the important factors in muscle mass degradation [28]. The ubiquitin-proteasome pathway, known to up-regulate MAFbx or MuRF1, results in muscle loss via disruption of homeostasis by catabolism [34]. The FOXO family is one of the transcription factors which controls the activity of MAFbx and MuRF1 expression [34]. The present study suggested that muscle loss was caused by the ubiquitin-proteasome pathway through FOXO3/MAFbx in a mouse model of dexamethasone-induced muscle loss and in C2C12 myoblasts. In addition, rutin inhibited the ubiquitin proteasome pathway and showed therapeutic efficacy in muscle loss models. The overall rutin mechanisms related to muscle loss are shown in Figure 7.

Sarcopenia is a representative muscle change that occurs with aging [35]. The necessity of preventing sarcopenia is increasing in the elderly because the severity of accompanying chronic diseases such as falls, fractures, and diabetes is increasing [36]. In addition, Due to associated chronic inflammation or neuromuscular junction degradation, it may be challenging to maintain muscle strength and muscle mass with proper exercise [37,38]. The present study showed that rutin could be a potential agent for muscle loss through the blocking ubiquitin-proteasome pathway associated with catabolic protein degradation. However, since this study is a preclinical study using cells and animals, it is unknown whether rutin has the same effect and mechanism on muscle loss in clinical practice. Therefore, further study through human clinical studies concerning rutin’s effect on human skeletal muscle loss is needed.

## 5. Conclusions

In this study, we showed that dexamethasone-induced muscle loss is due to catabolism by the ubiquitin-proteasome pathway. In addition, we showed that rutin blocks the FOXO3/MAFbx pathways to prevent catabolism through the ubiquitin-proteasome pathway. Therefore, rutin could be a potential agent for muscle loss such as sarcopenia through the blocking ubiquitin-proteasome pathway associated with catabolic protein degradation.

## Data Availability

All of the data is contained within the article.

## References

[1] Metodiewa D., Kochman A., Karolczak S. (1997). Evidence for Antiradical and Antioxidant Properties of Four Biologically Active N,N-Diethylaminoethyl Ethers of Flavaone Oximes: A Comparison with Natural Polyphenolic Flavonoid Rutin Action. IUBMB Life.

[2] Maleki S.J., Crespo J.F., Cabanillas B. (2019). Anti-Inflammatory Effects of Flavonoids. Food Chem..

[3] De Andrade Teles R.B., Diniz T.C., Costa Pinto T.C., de Oliveira Júnior R.G., Gama e Silva M., de Lavor É.M., Fernandes A.W.C., de Oliveira A.P., de Almeida Ribeiro F.P.R., da Silva A.A.M. (2018). Flavonoids as Therapeutic Agents in Alzheimer’s and Parkinson’s Diseases: A Systematic Review of Preclinical Evidences. Oxid. Med. Cell. Longev..

[4] Chun O.K., Lee S.G., Wang Y., Vance T., Song W.O. (2012). Estimated Flavonoid Intake of the Elderly in the United States and around the World. J. Nutr. Gerontol. Geriatr..

[5] Ross J.A., Kasum C.M. (2002). Dietary Flavonoids: Bioavailability, Metabolic Effects, and Safety. Annu. Rev. Nutr..

[6] Gullón B., Lú-Chau T.A., Moreira M.T., Lema J.M., Eibes G. (2017). Rutin: A Review on Extraction, Identification and Purification Methods, Biological Activities and Approaches to Enhance Its Bioavailability. Trends Food Sci. Technol..

[7] Bonechi C., Donati A., Tamasi G., Leone G., Consumi M., Rossi C., Lamponi S., Magnani A. (2018). Protective Effect of Quercetin and Rutin Encapsulated Liposomes on Induced Oxidative Stress. Biophys. Chem..

[8] Enogieru A.B., Haylett W., Hiss D.C., Bardien S., Ekpo O.E. (2018). Rutin as a Potent Antioxidant: Implications for Neurodegenerative Disorders. Oxid. Med. Cell. Longev..

[9] Yoo H., Ku S.-K., Baek Y.-D., Bae J.-S. (2014). Anti-Inflammatory Effects of Rutin on HMGB1-Induced Inflammatory Responses In Vitro and In Vivo. Inflamm. Res..

[10] Tian C., Guo Y., Chang Y., Zhao J., Cui C., Liu M. (2019). Dose-Effect Relationship on Anti-Inflammatory Activity on LPS Induced RAW 264.7 Cells and Antioxidant Activity of Rutin In Vitro. Acta Pol. Pharm. Drug Res..

[11] Lexell J., Taylor C.C., Sjöström M. (1988). What Is the Cause of the Ageing Atrophy? Total Number, Size and Proportion of Different Fiber Types Studied in Whole Vastus Lateralis Muscle from 15- to 83-Year-Old Men. J. Neurol. Sci..

[12] Lecker S.H., Solomon V., Mitch W.E., Goldberg A.L. (1999). Muscle Protein Breakdown and the Critical Role of the Ubiquitin-Proteasome Pathway in Normal and Disease States. J. Nutr..

[13] Sacheck J.M., Ohtsuka A., McLary S.C., Goldberg A.L. (2004). IGF-I Stimulates Muscle Growth by Suppressing Protein Breakdown and Expression of Atrophy-Related Ubiquitin Ligases, Atrogin-1 and MuRF1. Am. J. Physiol. Endocrinol. Metab..

[14] Tournadre A., Vial G., Capel F., Soubrier M., Boirie Y. (2019). Sarcopenia. Jt. Bone Spine.

[15] Senf S.M., Dodd S.L., Judge A.R. (2010). FOXO Signaling Is Required for Disuse Muscle Atrophy and Is Directly Regulated by Hsp70. Am. J. Physiol. Cell. Physiol..

[16] Yuan Y., Shi X., Liu Y., Yang G. (2011). FoxO1 Regulates Muscle Fiber-Type Specification and Inhibits Calcineurin Signaling during C2C12 Myoblast Differentiation. Mol. Cell. Biochem..

[17] Adams V., Gußen V., Zozulya S., Cruz A., Moriscot A., Linke A., Labeit S. (2020). Small-Molecule Chemical Knockdown of MuRF1 in Melanoma Bearing Mice Attenuates Tumor Cachexia Associated Myopathy. Cells.

[18] Foletta V.C., White L.J., Larsen A.E., Léger B., Russell A.P. (2011). The Role and Regulation of MAFbx/Atrogin-1 and MuRF1 in Skeletal Muscle Atrophy. Pflug. Arch..

[19] Bodine S.C., Baehr L.M. (2014). Skeletal Muscle Atrophy and the E3 Ubiquitin Ligases MuRF1 and MAFbx/Atrogin-1. Am. J. Physiol. Endocrinol. Metab..

[20] Seo S., Lee M.-S., Chang E., Shin Y., Oh S., Kim I.-H., Kim Y. (2015). Rutin Increases Muscle Mitochondrial Biogenesis with AMPK Activation in High-Fat Diet-Induced Obese Rats. Nutrients.

[21] Wang Q.-L., Huo X.-C., Wang J.-H., Wang D.-P., Zhu Q.-L., Liu B., Xu L.-L. (2017). Rutin Prevents the Ovariectomy-Induced Osteoporosis in Rats. Eur. Rev. Med. Pharmacol. Sci..

[22] Flavonoids: Nutraceutical Potential for Counteracting Muscle Atrophy|SpringerLink. https://link.springer.com/article/10.1007/s10068-020-00816-5.

[23] Shiota C., Abe T., Kawai N., Ohno A., Teshima-Kondo S., Mori H., Terao J., Tanaka E., Nikawa T. (2015). Flavones Inhibit LPS-Induced Atrogin-1/MAFbx Expression in Mouse C2C12 Skeletal Myotubes. J. Nutr. Sci. Vitaminol..

[24] Joshi V., Mishra R., Upadhyay A., Amanullah A., Poluri K.M., Singh S., Kumar A., Mishra A. (2019). Polyphenolic Flavonoid (Myricetin) Upregulated Proteasomal Degradation Mechanisms: Eliminates Neurodegenerative Proteins Aggregation. J. Cell. Physiol..

[25] Pallauf K., Duckstein N., Hasler M., Klotz L.-O., Rimbach G. (2017). Flavonoids as Putative Inducers of the Transcription Factors Nrf2, FoxO, and PPARγ. Oxid. Med. Cell. Longev..

[26] Zhao J., Brault J.J., Schild A., Cao P., Sandri M., Schiaffino S., Lecker S.H., Goldberg A.L. (2007). FoxO3 Coordinately Activates Protein Degradation by the Autophagic/Lysosomal and Proteasomal Pathways in Atrophying Muscle Cells. Cell Metab..

[27] Lundell L.S., Massart J., Altıntaş A., Krook A., Zierath J.R. (2018). Regulation of Glucose Uptake and Inflammation Markers by FOXO1 and FOXO3 in Skeletal Muscle. Mol. Metab..

[28] McCarthy J.J., Esser K.A. (2010). Anabolic and Catabolic Pathways Regulating Skeletal Muscle Mass. Curr. Opin. Clin. Nutr. Metab. Care.

[29] Lee S., Dong H.H. (2017). FoxO Integration of Insulin Signaling with Glucose and Lipid Metabolism. J. Endocrinol..

[30] Davila D., Connolly N.M.C., Bonner H., Weisová P., Dussmann H., Concannon C.G., Huber H.J., Prehn J.H.M. (2012). Two-Step Activation of FOXO3 by AMPK Generates a Coherent Feed-Forward Loop Determining Excitotoxic Cell Fate. Cell Death Differ..

[31] Gil da Costa R.M., Aragão S., Moutinho M., Alvarado A., Carmo D., Casaca F., Silva S., Ribeiro J., Sousa H., Ferreira R. (2017). HPV16 Induces a Wasting Syndrome in Transgenic Mice: Amelioration by Dietary Polyphenols via NF-ΚB Inhibition. Life Sci..

[32] Pomiès P., Blaquière M., Maury J., Mercier J., Gouzi F., Hayot M. (2016). Involvement of the FoxO1/MuRF1/Atrogin-1 Signaling Pathway in the Oxidative Stress-Induced Atrophy of Cultured Chronic Obstructive Pulmonary Disease Myotubes. PLoS ONE.

[33] Rom O., Reznick A.Z. (2016). The Role of E3 Ubiquitin-Ligases MuRF-1 and MAFbx in Loss of Skeletal Muscle Mass. Free Radic. Biol. Med..

[34] Price S.R., Bailey J.L., Wang X., Jurkovitz C., England B.K., Ding X., Phillips L.S., Mitch W.E. (1996). Muscle Wasting in Insulinopenic Rats Results from Activation of the ATP-Dependent, Ubiquitin-Proteasome Proteolytic Pathway by a Mechanism Including Gene Transcription. J. Clin. Investig..

[35] Volpi E., Nazemi R., Fujita S. (2004). Muscle Tissue Changes with Aging. Curr. Opin. Clin. Nutr. Metab. Care.

[36] Yeung S.S.Y., Reijnierse E.M., Pham V.K., Trappenburg M.C., Lim W.K., Meskers C.G.M., Maier A.B. (2019). Sarcopenia and Its Association with Falls and Fractures in Older Adults: A Systematic Review and Meta-analysis. J. Cachexia Sarcopenia Muscle.

[37] Bao Z., Cui C., Chow S.K.-H., Qin L., Wong R.M.Y., Cheung W.-H. (2020). AChRs Degeneration at NMJ in Aging-Associated Sarcopenia–A Systematic Review. Front. Aging Neurosci..

[38] Rudolf R., Khan M.M., Labeit S., Deschenes M.R. (2014). Degeneration of Neuromuscular Junction in Age and Dystrophy. Front. Aging Neurosci..

